# Supplementary material for: Probing the critical nucleus size in tetrahydrofuran clathrate hydrate formation using surface-anchored nanoparticles
Source: Nat Commun. 2024 Jan 2;15:157. doi: 10.1038/s41467-023-44378-6 (PMC10762117; doi:10.1038/s41467-023-44378-6)
Supplement: Supplementary file 1 — Supplementary information [file 41467_2023_44378_MOESM1_ESM.pdf]

Supplementary information for

Probing the critical nucleus size in tetrahydrofuran clathrate hydrate formation using surface-anchored nanoparticles

Han Xue[1]†, Linhai Li[1]†, Yiqun Wang[2], Youhua Lu[1], Kai Cui[1], Zhiyuan He[1], Guoying Bai[1], Jie Liu[1,3]*, Xin Zhou[2,4]*, Jianjun Wang[1,3,5]*

[1]Beijing National Laboratory for Molecular Science, Key Laboratory of Green Printing, Institute of Chemistry, Chinese Academy of Sciences, Beijing 100190, China.

[2]School of Physical Sciences, University of Chinese Academy of Sciences, Beijing 100049, China.

[3]School of Chemical Sciences, University of Chinese Academy of Sciences, Beijing 100049, China.

[4]Wenzhou Institute, University of Chinese Academy of Sciences, Wenzhou 325001, China.

[5]Technical Institute of Physics and Chemistry, Chinese Academy of Sciences, Beijing 100190, China.

†These authors contributed equally to this work.

*To whom correspondence should be addressed.

J. Wang (wangjianjun@mail.ipc.ac.cn), X. Zhou (xzhou@ucas.ac.cn), or J. Liu (liujie123@iccas.ac.cn)

This PDF file includes:

- Supplementary Figures (1-13)
- Supplementary Tables (1-5)
- Supplementary Discussion

Supplementary Figures (1-13)

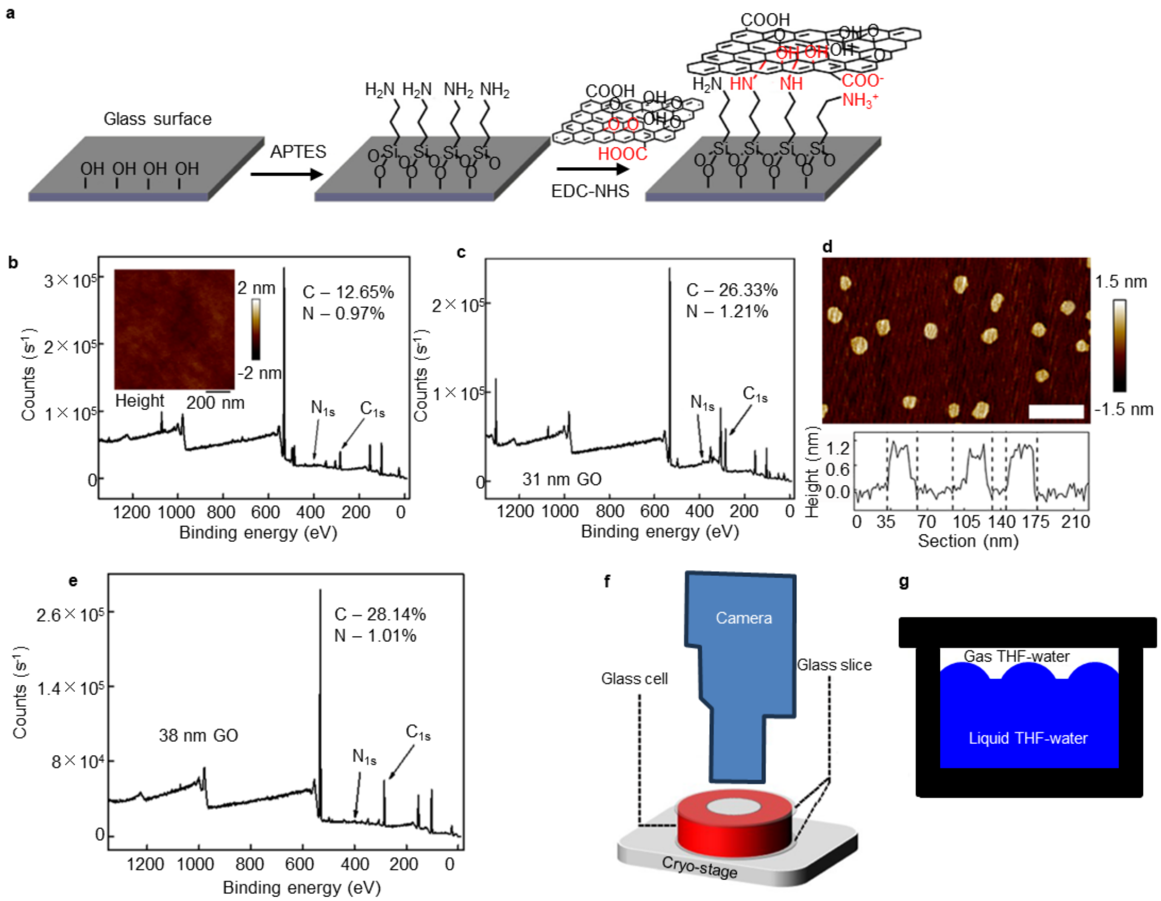

Supplementary Figure 1. Illustration of surface modification and setup of optical observation. a Proposed schematic illustration for grafting GOs on the glass substrate. **b** The elemental analysis from XPS spectrum of modified glass substrates with APTES, in which the appearance of N element indicating the APTES grafted. The inset shows the AFM characterizations of the prepared surfaces without GOs. **c** shows the chemical compositions of the glass substrate surfaces anchored with GOs of 38nm size. **d** shows the AFM imaging of 31 nm GOs and the corresponding height profiles along the blue marked lines. Scale bar, 100 nm. **e** shows the chemical compositions of the glass substrate surfaces anchored with GOs of 38nm size. From the XPS data, the coverage of anchored GOs can be calculated, in which the GO graft density can be adjusted through changing the reaction time. **f** The sketch of the THF/water sample cell, in which the contact area between the liquid sample and the solid surface can be well quantified through modulating the cell diameter. The clathrate nucleation was monitored through optical microscopy coupled with a high-speed CMOS camera. **g** The side view of experimental glass cell and illustration of gas THF-water between lid and liquid surface.

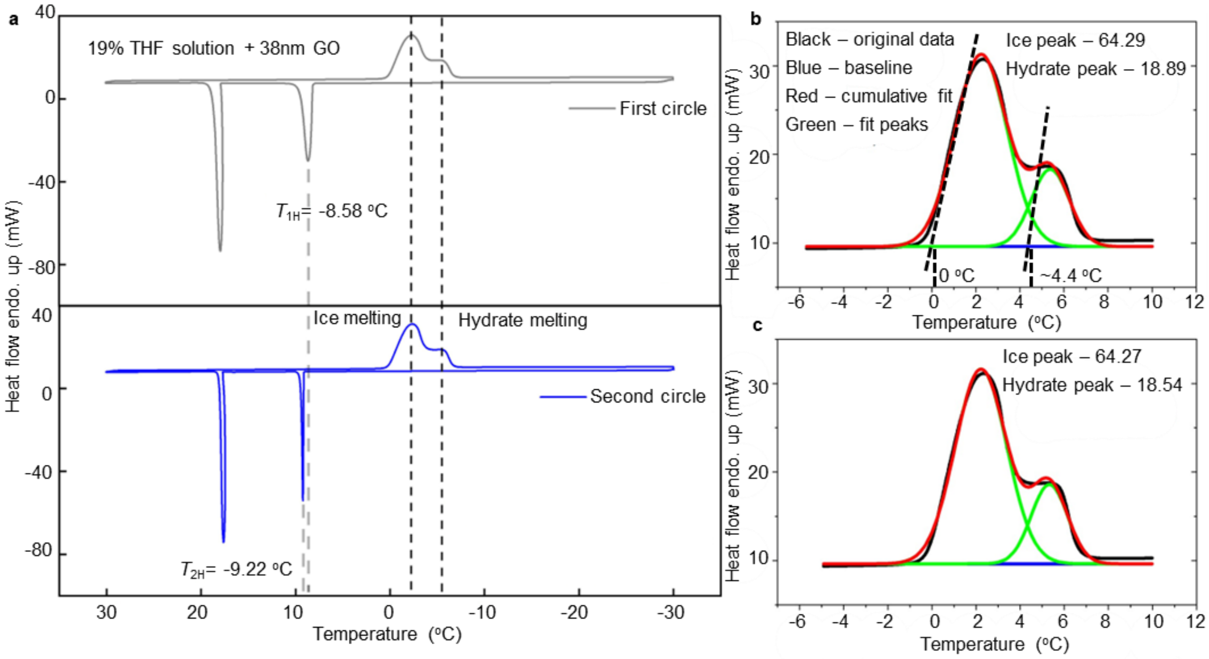

Supplementary Figure 2. DSC scans with two loops and excellent sealing. For showing the elimination of memory effect, we carried out DSC scans of the same sample with two loops shown in (**a**), in which the sample was kept at 30 °C for 30 minutes after the first-cycle scan (gray line), and then commencing the testing for the second-cycle scan (blue line). The results showed that the THF clathrate hydrate nucleation temperature in the second cycle is not increased. Thus, it indicates that our experimental setup of holding the sample at 30 °C for 30 minutes can eliminate the memory effect in the nucleation tests. Moreover, from (**b**) and (**c**), the calculation of formed ice (area of ice peaks) in the two loops are almost the same with the difference of 0.2%, and the formed THF hydrate are also almost the same with the difference of 1.5% (the red and black solid lines in the (**b**) and (**c**), respectively), indicating the excellent sealing in DSC scans.

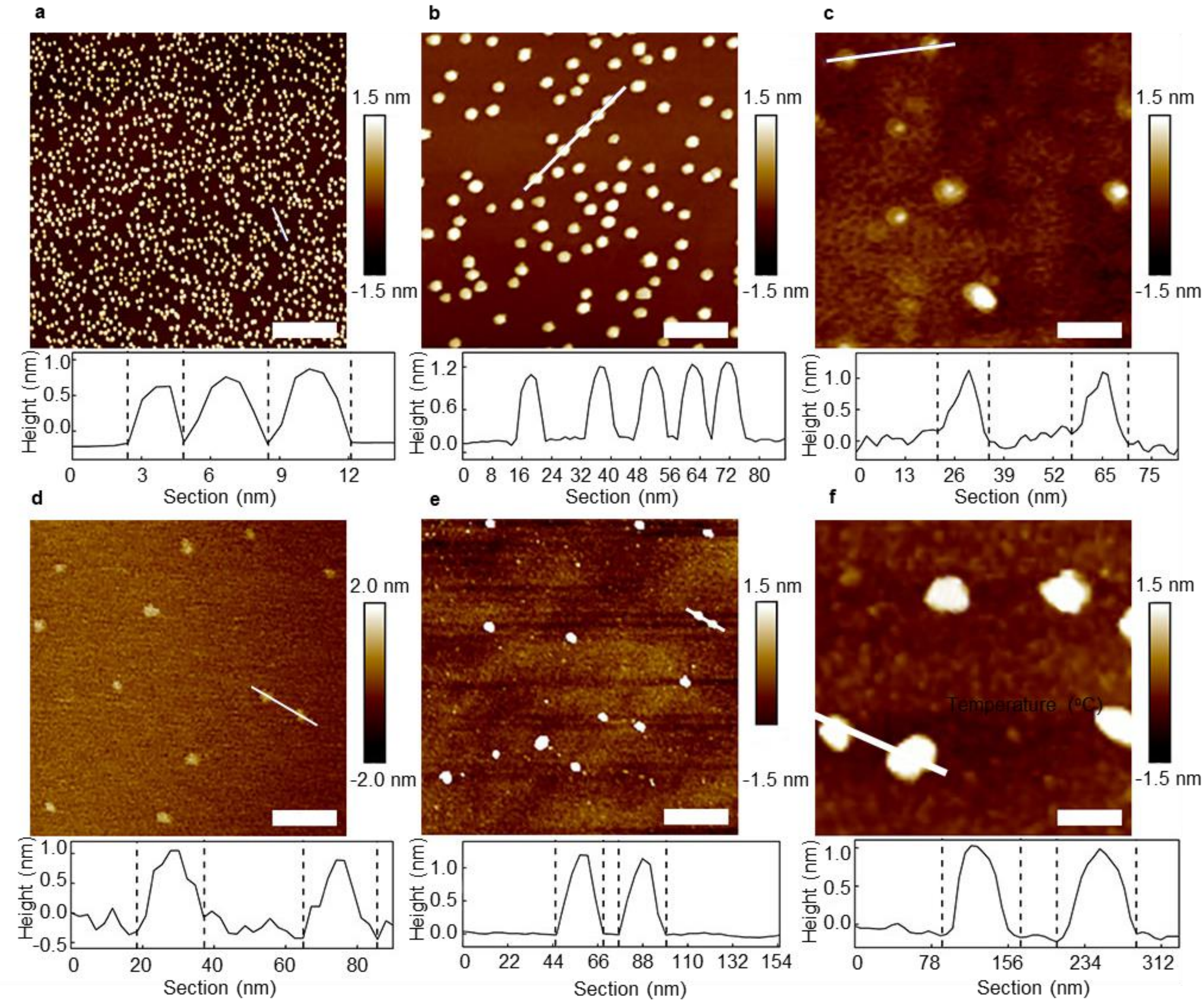

Supplementary Figure 3. Characterizations of GOs of controlled sizes. The GOs (**a**-3nm, **b**-8nm, **c**-13nm, **d**-22nm, **e**-46nm, **f**-78nm) were anchored on the glass surfaces, with AFM imaging and the corresponding height profiles along the white lines marked. (**a**) - (**c**), scale bar, 30 nm. (**d**) - (**f**), scale bar, 100 nm.

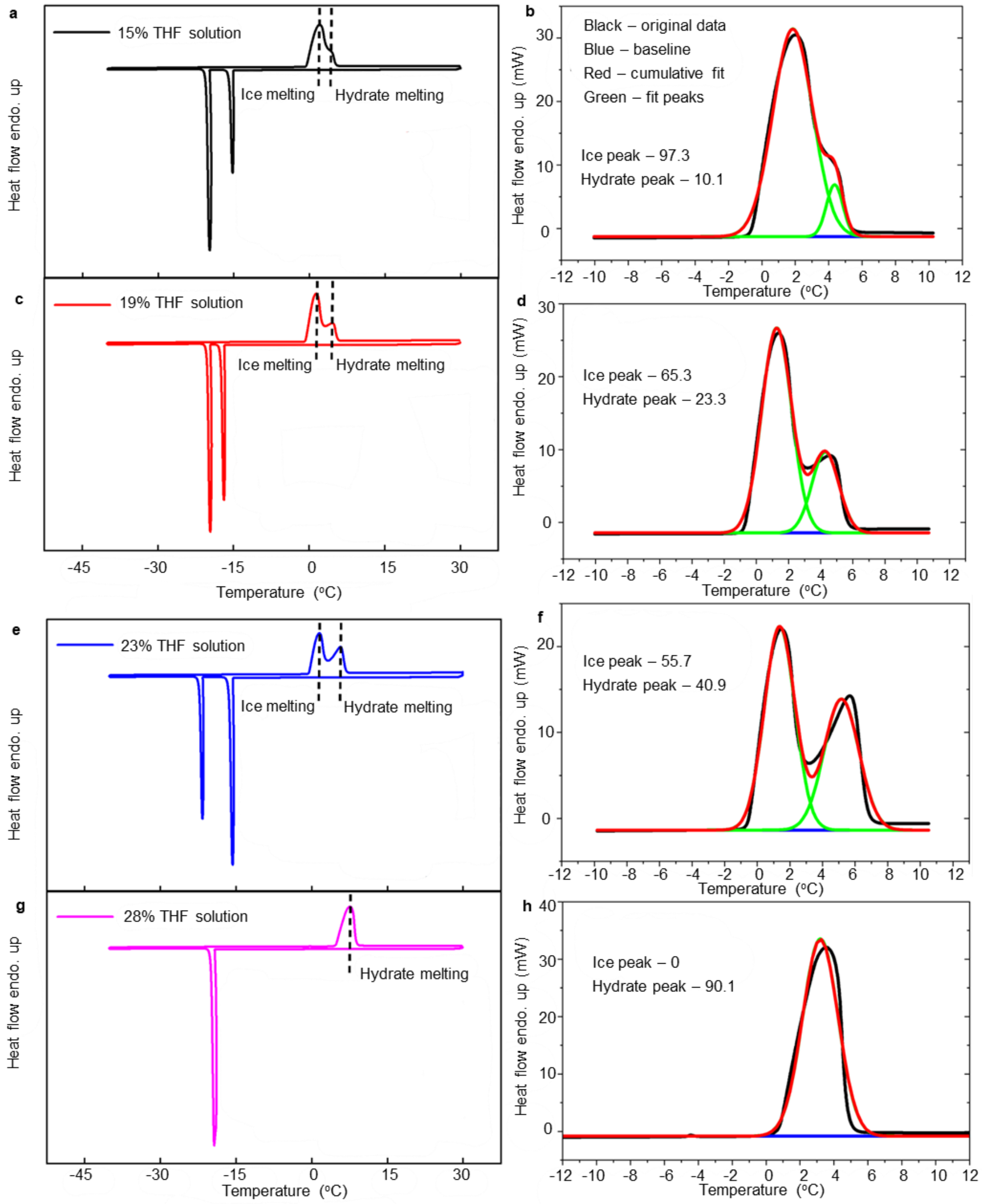

Supplementary Figure 4. Ice formation during cooling the THF solution with various THF/water ratio. **a** The typical DSC thermograph of THF mixed in water with 15 wt% in weight. **b** The melting peak analysis of formed ice and hydrate from (**a**) solution, respectively. **c** The typical DSC thermograph of THF mixed in water with 19 wt% in weight. **d** The melting peak analysis of formed ice and hydrate from (**c**) solution, respectively. **e** The typical DSC thermograph of THF mixed in water with 23 wt% in weight. **f** The melting peak analysis of formed ice and hydrate from (**e**) solution, respectively. **g** The typical DSC thermograph of THF mixed in water with 28 wt% in weight. **h** The melting peak analysis of formed ice and hydrate from (**g**) solution, respectively. The quantity of ice decreases as increasing the concentration, but reach zero until the concentration is a larger value than the expected 19%, (e.g., 28%), consisting with the argument that the remained water molecules inside the liquid droplet are excessed after the hydrate due to the evaporation of water molecules to the gas space less than the 17 times (the value in the clathrate) of that of THF molecules.

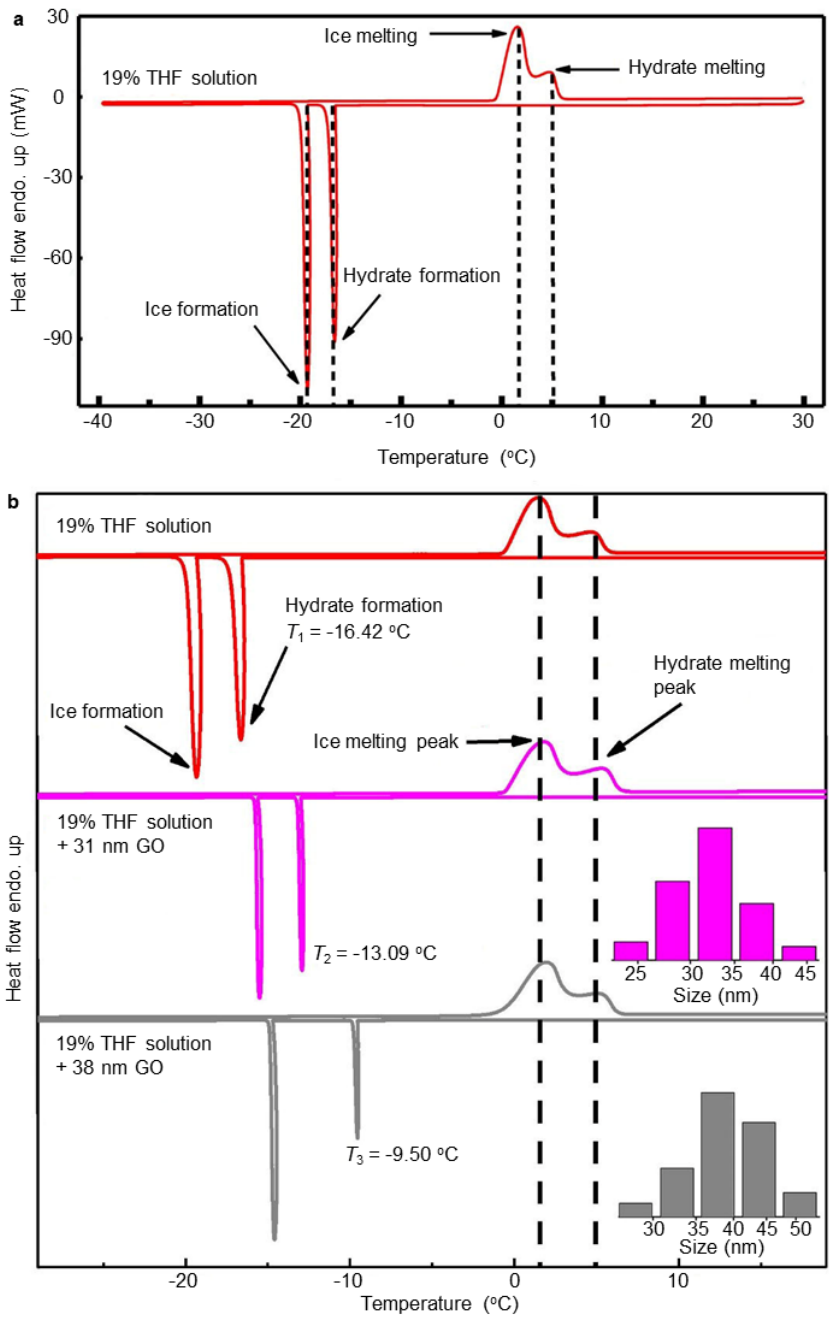

Supplementary Figure 5. Thermal analysis of the formation of THF clathrate hydrates. **a** The typical DSC thermograph of tetrahydrofuran (THF) mixed in water with 19 wt% in weight. The sample was first cooled to -40 ºC from 30 ºC and then heated to 30 ºC. The scan rate in the cooling step is 1 ºC min^{-1} and in the heating step is 5 ºC min^{-1}. In the cooling program, the formation of THF hydrate and ice can be distinguished from the two obvious crystallization peaks in the thermal curve, which also corresponding to the double melting peaks in the heating stage. **b** In the cooling program, the formation of THF hydrate and ice can be distinguished from the two obvious crystallization peaks in the thermal curve, which also corresponding to the double melting peaks in the heating procedure. The top red line shows that THF mixed in water with 19 wt%, the hydrate forming around T_1 = -16.42 ºC. The middle magenta line shows that the hydrate forming around T_2 = -13.09 ºC with adding GO nanosheets of 31 nm in the THF/water mixture. The bottom gray line shows that the hydrate formation enhanced about T_3 = -9.50 ºC with adding GOs of 38 nm in the THF/water mixture. The magenta and gray inset corresponding to the size distribution of 31 nm and 38 nm GOs, respectively. Sample volume is 3.0 µL. The scan rate of cooling curve is 1 ºC min^{-1} and the heating rate is 5 ºC min^{-1}.

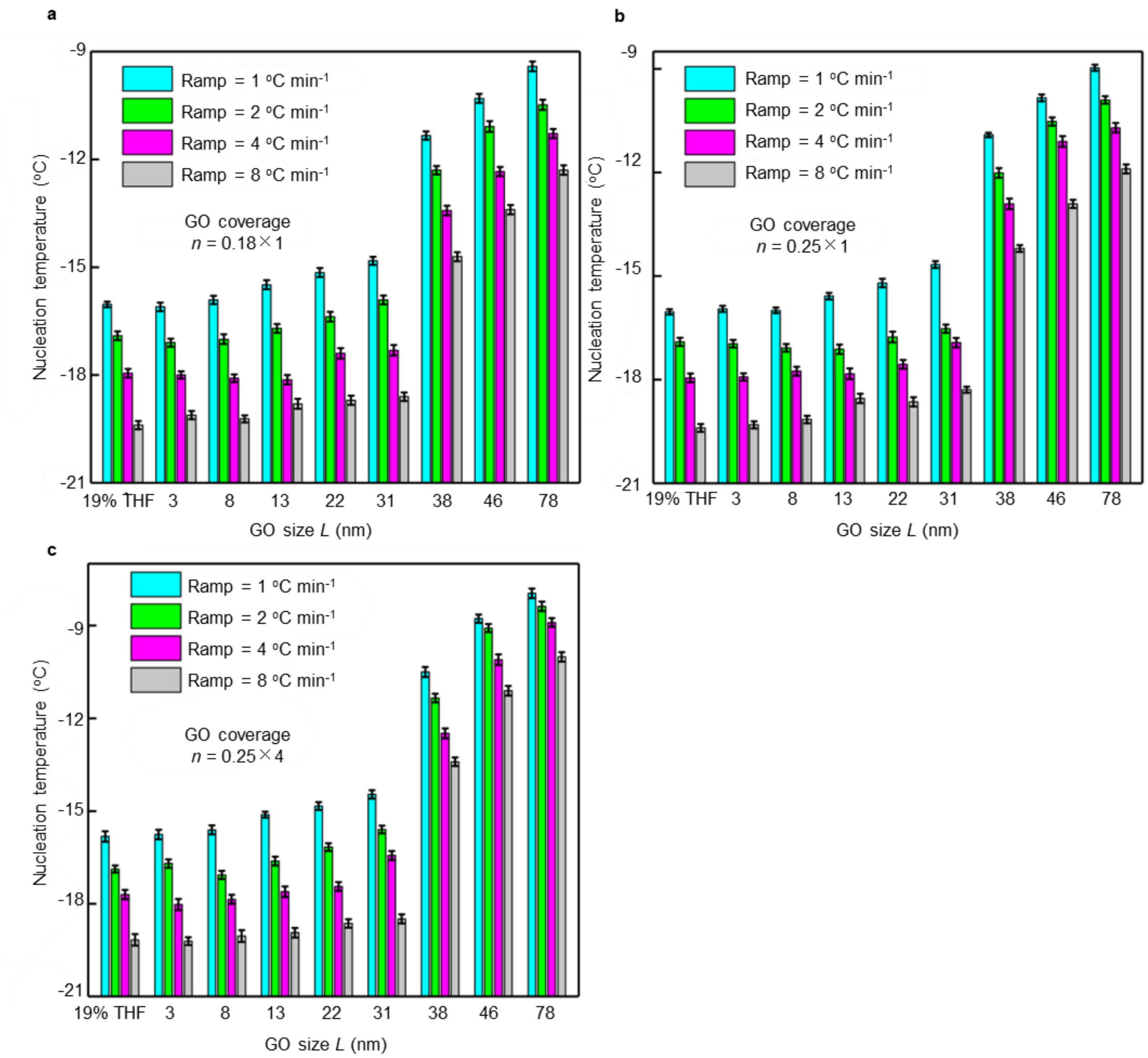

Supplementary Figure 6. Nucleation temperature of THF/water mixture containing GOs of controlled sizes. **a** The average nucleation temperature of THF clathrate tuned by GOs with various average sizes and different cooling rates, at the scaled GO coverage of n = 0.18. Each nucleation temperature on the substrate without GOs was averaged from 85 independent experiments. Error bars are the standard error of the mean (SEM). Data are shown as mean ± SEM. Each nucleation temperature on the substrate with GOs was averaged from 100 independent experiments. Data are mean ± SEM. **b** The average nucleation temperature of THF clathrate tuned by GOs with various average sizes and different cooling rates, at the scaled GO coverage of n = 0.25. Each nucleation temperature on the substrate without GOs was averaged from 85 independent experiments. Data are shown as mean ± SEM. Each nucleation temperature on the substrate with GOs was averaged from 100 independent experiments. Data are mean ± SEM. **c** The average nucleation temperature of THF clathrate tuned by GOs with various average sizes and different cooling rates, at the scaled GO coverage of n = 1. Each nucleation temperature on the substrate without GOs was averaged from 85 independent experiments. Data are shown as mean ± SEM. Each nucleation temperature on the substrate with GOs was averaged from 100 independent experiments. Data are mean ± SEM.

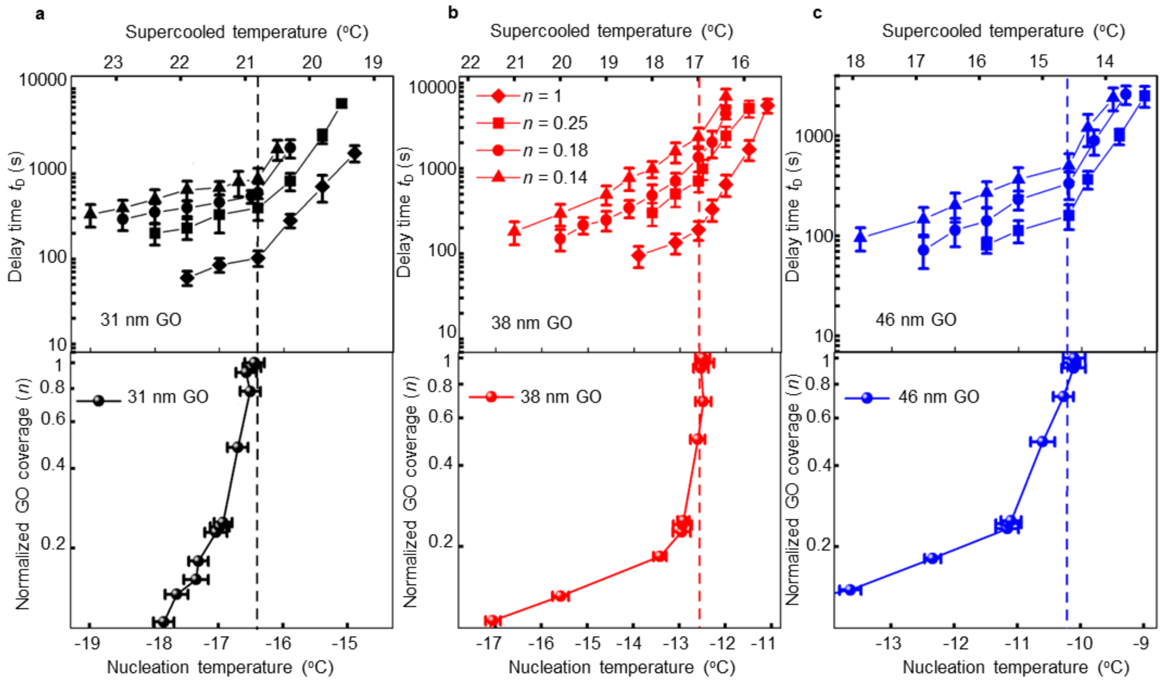

Supplementary Figure 7. The capability of GOs in facilitating the THF hydrate nucleation versus temperature. a The induction time of THF hydrate on GOs with coverage at lateral size of L = 31 nm, and the (supercooling) temperature occurring the hydrate nucleation versus the applied coverage of GOs. Every average clathrate hydrate nucleation delay time shows mean ± SEM. For the GO coverage of n = 0.14, the mean values were averaged from 36 measurements. For every other GO coverage, the mean values were averaged from 39 measurements. The clathrate hydrate nucleation delay time was independently measured with more than 35 valid nucleation events. **b** the induction time of THF hydrate on GOs with coverage at lateral size of L = 38 nm, and the (supercooling) temperature occurring the hydrate nucleation versus the applied coverage of GOs. The clathrate hydrate nucleation delay time was independently measured with more than 35 valid nucleation events. Every average clathrate hydrate nucleation delay time shows mean ± SEM. For the GO coverage of n = 0.14, the mean values were averaged from 36 measurements. For every other GO coverage, the mean values were averaged from 39 measurements. **c** the induction time of THF hydrate on GOs with coverage at lateral size of L = 46 nm, and the (supercooling) temperature occurring the hydrate nucleation versus the applied coverage of GOs. Every average clathrate hydrate nucleation delay time shows mean ± SEM. For the GO coverage of n = 0.14, the mean values were averaged from 36 measurements. For every other GO coverage, the mean values were averaged from 39 measurements. The dashed lines in (**a**) - (**c**) give ΔT_L for all the three-size GOs.

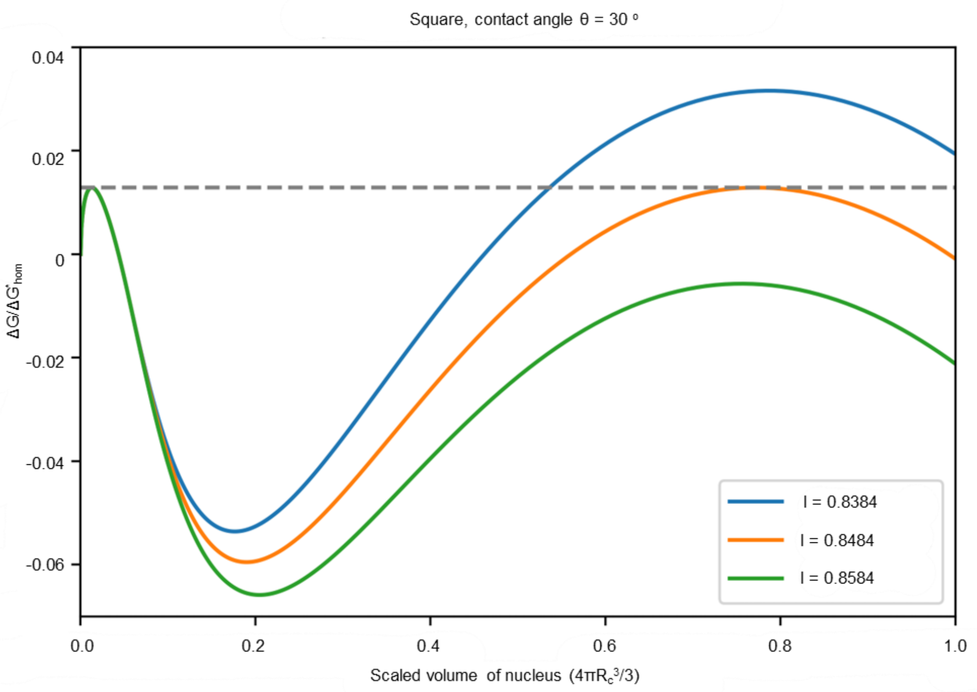

Supplementary Figure 8. The relationship between nuclei size and free energy. The illustrated free energies (in the unit of the homogeneous nucleation free energy barrier) versus the volume of nucleus (in the unit of $4\pi/3{R_c}^3$) on nanocubes with the lateral size $L \sim 2R_c$ and strong ability on facilitating the nucleation (contact angle between substrate and clathrate nuclei as 30 degree). The results are calculated numerically via the software Surface Evolver to get the optimized nuclei sitting on squared substrates (nanocubes) with the contact angle $\theta = 30^o$. There are two free barriers of nucleation on the finite-size nanocubes with the dimensionless size $l \equiv \frac{L}{2R_c} \sim l_c$. For small nanocubes ($l < l_c$), the second barrier dominates the occurrence of nucleation, but for large nanocubes ($l < l_c$), the first one which is the same as that of the normal heterogeneous nucleation on infinite surfaces dominates the nucleation; and the two barriers are equal giving that $l = l_c$. Here $l_c \approx 0.8136$, which is slightly dependent on the interaction between nanocubes and nucleus, *i.e.*, the contact angle, θ.

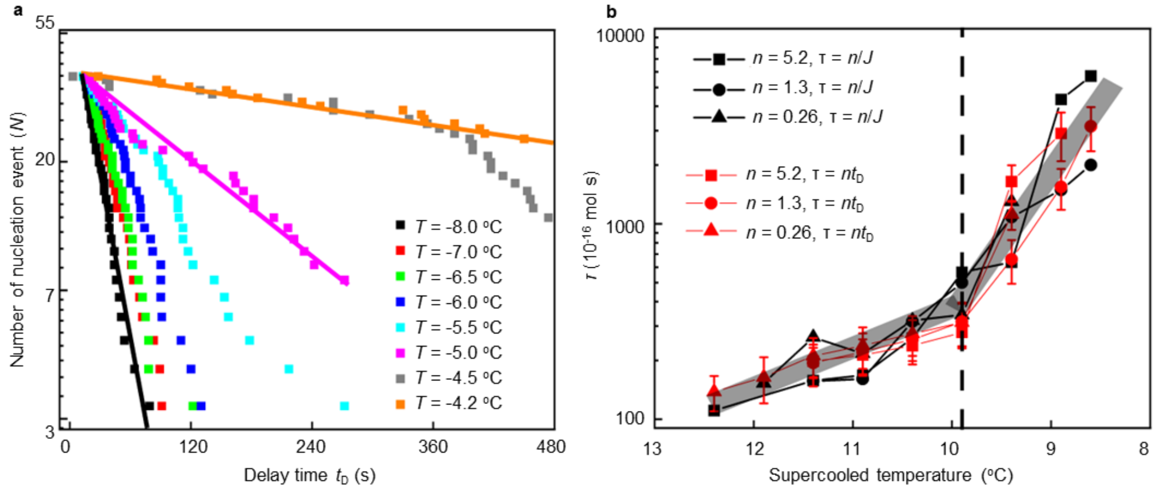

Supplementary Figure 9. Nucleation rates (*J*) of the THF aqueous solution at different temperatures. a The unfrozen droplets of the THF aqueous solution versus the delay time (t_D) on the 45 nm Au nanocubes anchored surfaces of $n = 5.2 \times 10^{-16}$ mol. **b** $\tau = nt_D$ (T; n) (in red) and $\tau = n/J$ (J; n) (in black) versus ΔT. *J* is the nucleation rate calculated from the freezing shown in (**a**). Every average clathrate hydrate nucleation delay time shows mean ± SEM. The mean value was averaged from 36 measurements for 45 nm Au nanocube with coverage of $n = 0.26$. For the every other nanocube coverage, the mean values were averaged from 39 measurements.

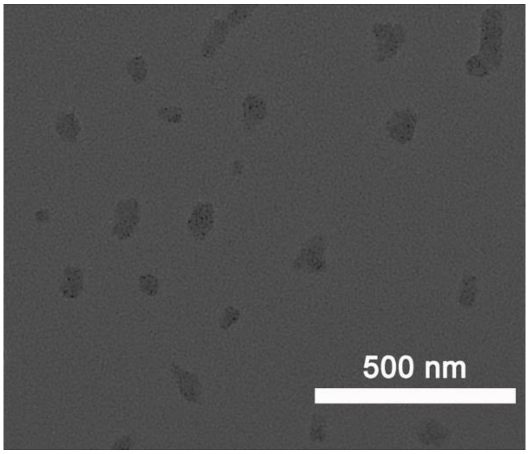

Supplementary Figure 10. TEM images of raw GO materials before the size fraction classification.

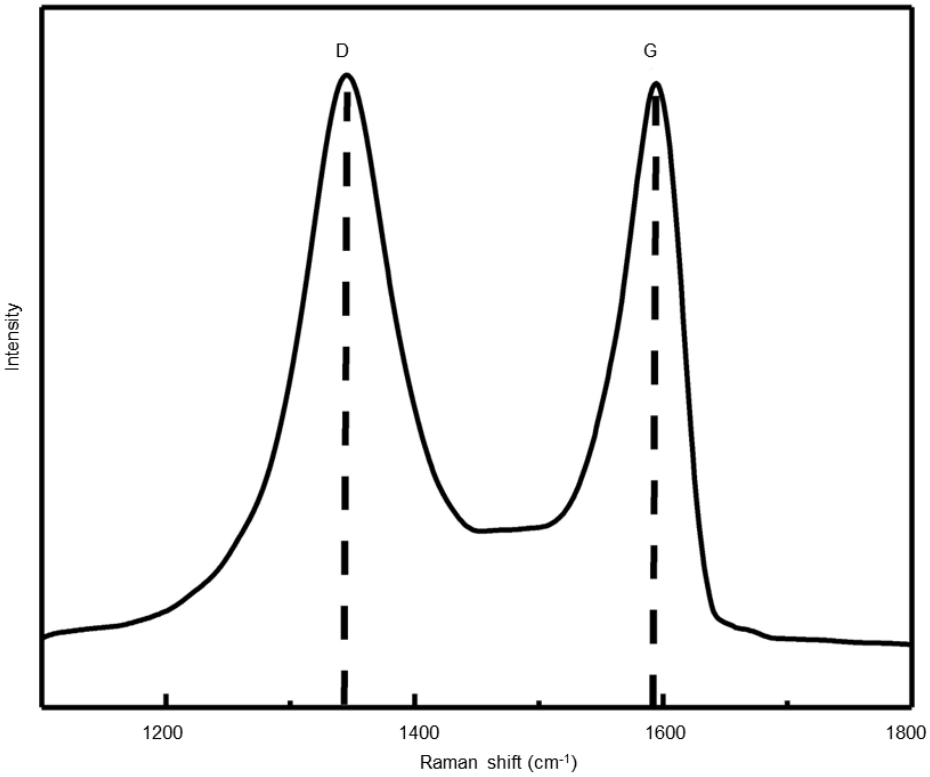

Supplementary Figure 11. Raman spectrum of raw GO materials before the size fraction classification. The most prominent features in the Raman spectra of GO materials are the so-called G band appearing at about 1594 cm^{-1}, the D band at about 1350 cm^{-1}. The integrated intensity ratio I_D/I_G for the D band and G band is widely used for characterizing the defect quantity in GO materials. The I_D/I_G of raw GOs in this study is calculated of 1.04. This indicates that the GO nanosheets are typical and have been used in previous publications.

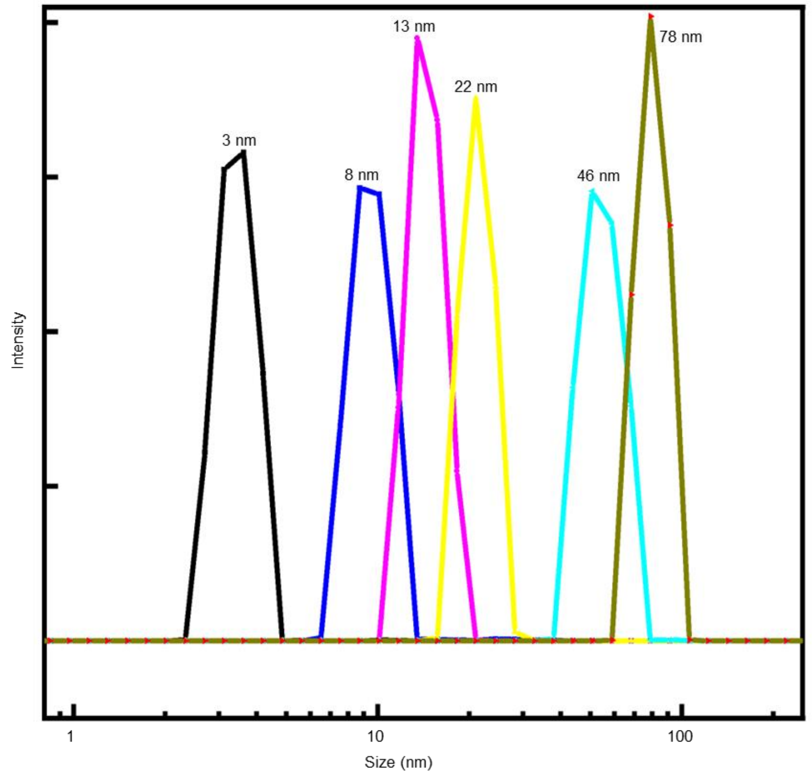

Supplementary Figure 12. Hydrodynamic diameter distributions of GOs with controlled sizes. Typical hydrodynamic diameter distributions of various size-fractioned GO samples measured by dynamic light scattering spectrometer. The results confirm GOs of controlled sizes.

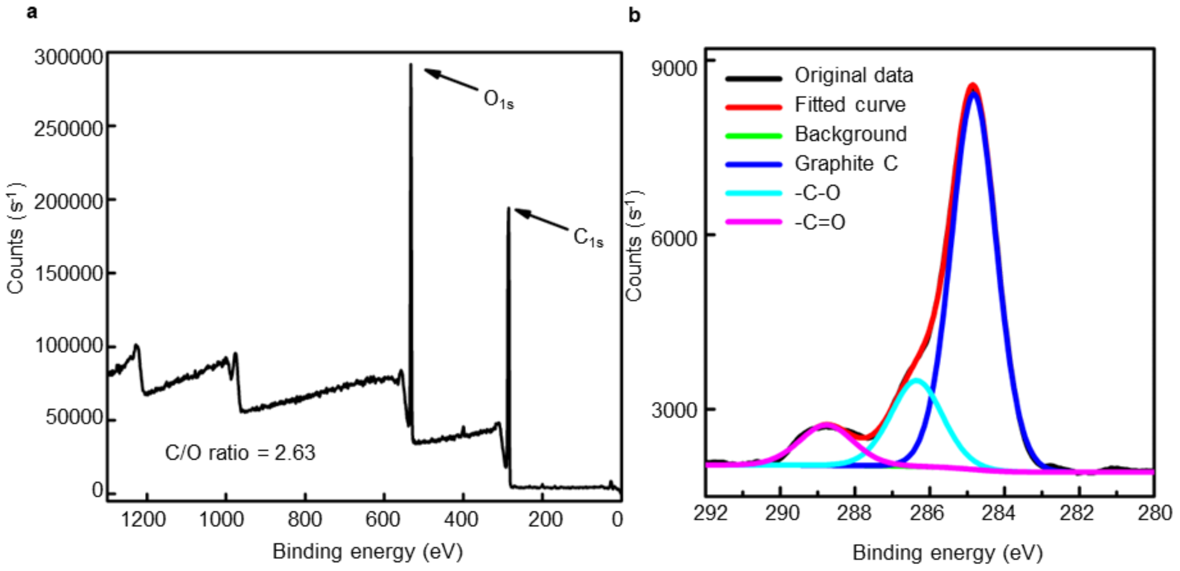

Supplementary Figure 13. X-ray photoelectron spectroscopy (XPS) spectrum of raw GO materials before the size fraction classification. a The elementary analysis indicates the good purity of raw GOs. From the spectrum, the content only consisting of carbon and oxygen. **b** shows the C1s core-level XPS spectrum of raw GOs with the C/O ratio is calculated of 2.63. This indicates that the GO nanosheets are typical and have been used in previous publications.

Supplementary Tables (1-5)

Time	Element N (%)	Element C (%)
0	0	0
15 min	0.82±0.06	10.45±0.86
30 min	0.91±0.04	10.49± 0.77
45 min	0.96±0.03	11.12±0.64
3 h	1.35±0.04	11.86±0.84
7 h	1.43±0.02	12.42±0.85
18 h	2.98±0.05	25.77±0.91

Supplementary Table 1. The amount of nitrogen and carbon element on APTES vs the grafting reaction time. The results show mean ± s.e.m. from 3 samples.

Time	Element N (%)	Element C (%)
0	0.96±0.03	11.12±0.64
1 h	1.01±0.08	14.77±0.59
2 h	1.09±0.09	16.69±0.97
4 h	1.14±0.10	17.31±0.60
6 h	1.07±0.08	17.40±1.16
8 h	1.27±0.02	17.78±1.02
10 h	1.23±0.05	17.88±0.97
12 h	1.13±0.11	18.82±1.09
14 h	1.07±0.10	21.24±1.74
16 h	1.09±0.09	22.13±1.40
18 h	1.14±0.06	22.71±1.36
24 h	1.15±0.10	26.64±1.16

Supplementary Table 2. The amount of nitrogen and carbon element on APTES vs the grafting reaction time of GOs with the average size 38 nm. The results show mean ± s.e.m. from 3 samples.

Time	Element N (%)	Element C (%)
0	0.96±0.03	11.12±0.64
4 h	1.04±0.07	17.11±0.76
12 h	1.24±0.05	20.48±0.93
24 h	1.31±0.07	27.83±0.67

Supplementary Table 3. The amount of nitrogen and carbon element on APTES vs the grafting reaction time of GOs with the average size 31 nm. The results show mean ± s.e.m. from 3 samples.

C content (%)	ΔC content (%)	Max ΔC (%)	Relative density (%)
11.12±0.64	0	15.52	0
13.03±0.93	1.91	15.52	12.33±0.93
14.20±0.80	3.08	15.52	19.85±0.80
15.06±0.76	3.94	15.52	25.36±0.76
16.96±0.90	5.84	15.52	37.62±0.90
19.26±1.22	8.14	15.52	52.48±1.22
22.55±1.03	11.43	15.52	73.65±1.03
25.21±0.69	14.09	15.52	90.79±0.69
26.07±0.76	14.95	15.52	96.34±0.76
26.64±1.16	15.52	15.52	100

Supplementary Table 4. The relative grafting densities of 38 nm GOs anchored on substrates, calculated by ΔC/Max ΔC. The results show mean ± s.e.m. from 3 samples.

C content (%)	ΔC content (%)	Max ΔC (%)	Relative density (%)
11.12±0.64	0	16.71	0
12.51±0.60	1.39	16.71	8.34±0.60
14.32±0.62	3.20	16.71	19.15±0.62
18.16±0.81	7.04	16.71	42.12±0.81
20.06±0.81	8.94	16.71	53.52±0.81
21.29± 0.60	10.17	16.71	60.88±0.60
23.07±0.72	11.95	16.71	71.5±0.72
26.43±1.02	15.31	16.71	91.65±1.02
27.18±0.86	16.06	16.71	96.12±0.86
27.83±1.10	16.71	16.71	100

Supplementary Table 5. The relative grafting densities of 31 nm GOs anchored on substrates. The results show mean ± s.e.m. from 3 samples.

Supplementary Discussion

Differential scanning calorimetry (DSC) was the direct calorimetric measurement used to study the formation of THF hydrates. One typical DSC thermograph of 19 wt% THF in water solution is illustrated in Supplementary Figure 5a. When the sample is cooled to -40 ºC with rate of 1 ºC min^{-1} (see Methods), there are two exothermic peaks beginning at about -14 °C and -17 ºC, which distinguished to be assigned as hydrate and ice formation, respectively. Once all of the sample is frozen at -40 ºC, the final heating with rate of 5 ºC min^{-1} produces two peaks: the ice peak at 0 °C and the hydrate peak at about 4.4 °C, which is in fair agreement with the published value. The middle row in Supplementary Figure 5b shows the DSC thermographs obtained when THF/water with 31 nm (average lateral size) GOs is cooled to -40 ºC and then heated. It is observed that both of hydrate and ice are formed at higher nucleation temperature compared with the pure THF aqueous solution (Supplementary Figure 5a) under the experimental conditions. This indicates that the GOs can facilitate the formation of hydrate and ice, which is consistent with the previous results. Moreover, the addition of GO nanosheets with 38 nm can trigger the hydrate nucleation with a much higher temperature. This demonstrates that the size of GO can affect the hydrate nucleation, as the larger GO nanosheets facilitate the clathrate formation. Furthermore, the nucleation of hydrate always occurs before the ice with cooling the experimental sample due to the equilibrium melting temperature of THF hydrate is higher than ice, as shown in Fig. 1. It illustrates that clathrates cannot heterogeneously nucleate ice, which agree with previous reports.
