# Supplementary material for: Probing the critical nucleus size in tetrahydrofuran clathrate hydrate formation using surface-anchored nanoparticles
Source: Nat Commun. 2024 Jan 2;15:157. doi: 10.1038/s41467-023-44378-6 (PMC10762117; doi:10.1038/s41467-023-44378-6)
Supplement: Supplementary file 3 — Description of Additional Supplementary Files [file 41467_2023_44378_MOESM3_ESM.pdf]

Description of Additional Supplementary Files

File Name: Supplementary Movie 1

Description: The formation of THF clathrate crystal from solution is determined by the consecutive optical microscopic observation.
